# Supplementary material for: The Gut Microbiome in Stevens–Johnson Syndrome and Sjögren’s Disease: Correlations with Dry Eye
Source: Microorganisms. 2025 Nov 29;13(12):2730. doi: 10.3390/microorganisms13122730 (PMC12735903; doi:10.3390/microorganisms13122730)
Supplement: Supplementary file 1 [file microorganisms-13-02730-s001.zip › Table S1.pdf]

Table S1

SJS Group

Patient	HCQ	Omega-3	Ocular corticosteroids	Ocular Tacrolimus	Oral corticosteroids	Oral Immunosuppressants	Others
1	-	-	-	-	-	-	Warfarin
2	-	Yes	-	-	-	-	Aspirin, Losartan, Amlodipine
3	-	Yes	-	Yes	-	-	-
4	-	-	Fluticasone	Yes	-	-	Valproic Acid, Atenolol
5	-	-	-	Yes	-	-	-
6	-	-	-	-	-	-	-
7	-	-	-	Yes	-	-	Levothyroxine
8	-	-	Fluticasone	-	-	-	-
9	-	-	-	-	-	-	-

SjD Group

Patient	HCQ	Omega-3	Ocular corticosteroids	Ocular Tacrolimus	Oral corticosteroids	Oral Immunosuppressants	Others
1	5	Yes	-	-	Predisone 20mg	-	-
2	8	-	-	-	-	Leflunomide	-
3	12	-	-	-	-	-	Enalapril, Atenolol
4	3	-	-	-	-	-	Vitamin D, Gabapentin
5	8	Yes	-	-	-	-	Synthroid
6	5	Yes	-	-	-	-	-
7	11	Yes	-	-	-	-	-
8	2	Yes	-	-	-	-	Gabapentin
9	3	Yes	-	-	-	Azathioprine	-
10	8	Yes	-	-	-	Metrotrexate	-
