# Supplementary material for: The Gut Microbiome in Stevens–Johnson Syndrome and Sjögren’s Disease: Correlations with Dry Eye
Source: Microorganisms. 2025 Nov 29;13(12):2730. doi: 10.3390/microorganisms13122730 (PMC12735903; doi:10.3390/microorganisms13122730)
Supplement: Supplementary file 1 [file microorganisms-13-02730-s001.zip › Table S2.pdf]

Table S2

SJS Phylum	*r*	*p* value Without correction[1]	With correction[2]
**Actinobacteriota**			
DED DEWS	−0,173	0,656	0,835
NEI Score	−0,695	0,038	0,189
Schirmer test	0,167	0,668	0,835
TBUT	−0,353	0,351	0,835
OSDI	−0,033	0,932	0,932
**Synergistota**			
DED DEWS	−0,593	0,092	0,154
NEI Score	−0,722	0,028	0,140
Schirmer test	0,593	0,092	0,154
TBUT	−0,196	0,614	0,614
OSDI	−0,479	0,192	0,240
**SJS genus**			
**Christensenellaceae**			
DED DEWS	−0,696	0,037	0,187
NEI Score	0,042	0,915	0,915
Schirmer test	0,536	0,137	0,343
TBUT	0,451	0,223	0,371
OSDI	−0,050	0,898	0,915
**Subdoligranulum**			
DED DEWS	−0,317	0,407	0,678
NEI Score	−0,690	0,039	0,197
Schirmer test	0,339	0,371	0,678
TBUT	−0,057	0,884	0,884
OSDI	−0,226	0,558	0,698
**Clostridia**			
DED DEWS	−0,440	0,235	0,372
NEI Score	0,392	0,297	0,372
Schirmer test	0,407	0,277	0,372
TBUT	0,803	0,009	0,045
OSDI	0,102	0,795	0,795

SjD genus	*r*	*p* value Without correction[1]	With correction[2]
**Clostridia**			
DED DEWS	−0,684	0,029	0,145
NEI Score	−0,445	0,197	0,303
Schirmer test	0,407	0,243	0,303
TBUT	0,273	0,446	0,446
OSDI	−0,552	0,098	0,246
